# circPVT1 regulates medullary thyroid cancer growth and metastasis by targeting miR-455-5p to activate CXCL12/CXCR4 signaling

**DOI:** 10.1186/s13046-021-01964-0

**Published:** 2021-05-07

**Authors:** Xun Zheng, Shu Rui, Xiao-Fei Wang, Xiu-He Zou, Yan-Ping Gong, Zhi-Hui Li

**Affiliations:** Department of Thyroid and Parathyroid Surgery Center, West China Hospital, Sichuan University, No. 37 Guo Xue Xiang, 610041 Chengdu, Sichuan China

**Keywords:** Medullary thyroid cancer, miR-455-5p, circPVT1, CXCL12/CXCR4 signaling pathway

## Abstract

**Background:**

Medullary thyroid cancer (MTC) represents 13.4 % of all thyroid cancers-related deaths. The treatments for MTC are very limited especially for patients with distal metastasis. Therefore, it is critical to understand the mechanisms of MTC to pursue novel therapeutic avenues. Here, we studied the function of circPVT1/miR-455-5p in MTC.

**Methods:**

Human MTC tissues and cell lines were used. qRT-PCR and Western blotting were employed to measure expression levels of miR-455-5p, circPVT1, CXCL12, and epithelial mesenchymal transformation (EMT)-related proteins. Colony formation assay, flow cytometry, transwell assay, and scratch wound healing assay were used to assess the abilities of cell proliferation, apoptosis, migration and invasion, respectively. Dual luciferase assay and RNA immunoprecipitation were employed to validate interactions of circPVT1/miR-455-5p and miR-455-5p/CXCL12. Nude mouse xenograft model was used to evaluate the effects of shcircPVT1 and miR-455-5p mimics on tumor growth and metastasis *in vivo*.

**Results:**

miR-455-5p was reduced in MTC tissues and cells while circPVT1 was elevated. Their levels were correlated with prognosis of MTC. Overexpression of miR-455-5p or sh-circPVT1 suppressed EMT and MTC cell proliferation, migration and invasion. miR-455-5p targeted CXCL12 while circPVT1 sponged miR-455-5p. Knockdown of CXCL12 or CXCL12/CXCR4 signaling inhibitor reversed the effects of circPVT1 overexpression or miR-455-5p inhibitor on EMT and MTC cell proliferation, migration and invasion. Knockdown of circPVT1 or miR-455-5p overexpression repressed MTC tumor growth and lung metastasis *in vivo*.

**Conclusions:**

miR-455-5p suppresses MTC growth and metastasis by targeting CXCL12/CXCR4 signaling pathway while circPVT1 promotes MTC by sponging miR-455-5p. Our study sheds light on the mechanisms of MTC growth and metastasis.

**Supplementary Information:**

The online version contains supplementary material available at 10.1186/s13046-021-01964-0.

## Background

Medullary thyroid cancer (MTC) is a rare neuroendocrine cancer and represents about 3–5 % of all thyroid carcinomas [1, 2]. It stems from calcitonin-producing parafollicular C cells. About one quarter of MTC cases are inherited as familial syndrome or as a component of the multiple endocrine neoplasia 2 (MEN2) syndromes caused by activating germline mutations of the Rearranged during Transfection (RET) proto-oncogene, and the rest arise spontaneously and sporadically [3, 4]. The current treatments to MTC, to date, include radical thyroidectomy with or without lymphadenectomy [5, 6]. However, due to the lack of early diagnosis, most patients are diagnosed and receive treatments at late stages when regional or distal metastases have occurred and surgical resection is insufficient [1, 2]. Therefore, searching for new therapeutic treatments is necessary for better outcomes and it requires a good understanding of the pathogenesis of MTC.

MicroRNAs (miRNAs) are a well-known class of endogenous non-coding RNAs that play critical roles in varieties of cellular processes such as cell growth, proliferation, and differentiation [7, 8]. In addition, dysregulated miRNA levels have been implicated in many diseases including cancers [9]. In MTC, many aberrant expressions of miRNAs have been reported and one of them is miR-455-5p [10]. miR-455-5p has been shown to function as a tumor suppressor in many types of cancers such as colorectal cancer and prostate cancer [11, 12]. For instance, miR-455-5p induced cell apoptosis and suppressed cancer cell migration and invasion in hepatocellular carcinoma via targeting IGF-1R [13]. A study shows that miR-455-5p is down-regulated in MTC tissues [14], suggesting that it is involved in MTC. Nevertheless, the exact function of miR-455-5p in MTC remains largely unknown.

Circular RNAs (circRNAs) are a relatively novel class of endogenous non-coding RNAs [15, 16]. They are covalently closed and have very stable structure [16]. Emerging evidence indicates that circRNAs play important roles in many processes including normal physiological processes and abnormal pathological processes [16, 17]. The majority of circRNAs function by binding to miRNAs and act as miRNA sponges. circPVT1 is a key biomarker for many cancers and many studies have shown that circPVT1 has critical roles in mediating the cancer development[18, 19]. In esophageal carcinoma, circPVT1 level was increased and overexpression of circPVT1 promoted proliferation and invasion of cancer cells [18]. Similarly, elevated level of circPVT1 was reported in oral squamous cell carcinoma and it facilitated cancer cell proliferation by sponging miR-125b [20]. Nevertheless, it remains largely unknown whether circPVT1 is involved in MTC. Interestingly, through our preliminary bioinformatic analysis, we found some complementary bindings sites between circPVT1 and miR-455-5p. Further, we also identified some binding sites between miR-455-5p and CXCL12 (C-X-C chemokine ligand 12) mRNA. CXCL12 is a chemokine and binds to CXCR4 (C-X-C chemokine receptor 4) [21]. CXCR4 is widely expressed in many tissues and CXCL12/CXCR4 signaling plays an important role in diverse processes such as cell proliferation, survival, and differentiation, as well as inflammatory responses and tumor development [21]. Previous studies have shown that CXCR4 is elevated in MTC tissues and CXCL12/CXCR4 signaling promotes cancer cell proliferation and invasion [22]. Based on the aforementioned studies and our preliminary analysis, we hypothesized that circPVT1/miR-455-5p might be involved in MTC through CXCL12/CXCR4 signaling.

In the present study, we sought to investigate the function and underlying mechanism of circPVT1/miR-455-5p in the development of MTC. Using human MTC tissues and MTC cells lines, we found that miR-455-5p was reduced while circPVT1 was increased in both MTC tissues and cells. Overexpression of miR-455-5p or knockdown of circPVT1 suppressed MTC cell proliferation, migration, and invasion. Further they inhibited MTC tumor growth and lung metastasis *in vivo*. Mechanistically, we showed that circPVT1 directly bound miR-455-5p and miR-455-5p targeted CXCL12. miR-455-5p regulated MTC cell proliferation and metastasis via CXCL12/CXCR4 signaling and circPVT1 acted as a miR-455-5p sponge. Together, our study reveals a crucial role of circPVT1/miR-455-5p/CXCL12 axis in MTC development. These results help to shed light on the molecular mechanisms of MTC growth and metastasis and provide targets for the development of future therapy for MTC.

## Materials and methods

### Human MTC samples

Human MTC tissues were collected from 28 diagnosed MTC patients during surgical resection from West China Hospital of Sichuan University. The adjacent non-tumor medullary thyroid tissues were collected as control samples. The patients did not receive preoperative treatments before the collection. The procedure and protocol have been reviewed and approved by the ethics committee of West China Hospital of Sichuan University. All patients were informed of the study and signed the written consent. All human specimens were snap-frozen immediately in liquid nitrogen and kept for further experiments at -80 °C.

### Cell culture

Two human MTC cell lines (TT, MZ-CRC-1), and one normal human thyrocyte cell line (NThy-ori3.1) were used for the study as TT and MZ-CRC-1 are the two of most widely used MTC-derived cell lines [23–25]. They were obtained from the Cell Bank of the Chinese Academy of Sciences (Shanghai, China). The medium used to grow the cells was made of Dulbecco’s Modified Eagle Medium (DMEM) (Gibco, CA, USA), 10 % fetal bovine serum (ThermoFisher Scientific, MA, USA) and 1 % penicillin-streptomycin (P/S). Cells were maintained at 37 °C in standard cell incubator.

### Cell transfection

The full length of circPVT1 was cloned into the overexpression construct (pcDNA3.1). miR-455-5p mimics and inhibitor were synthesized and purchased from Genepharma (Shanghai, China). Sh-circPVT1-1, sh-circPVT-2, sh-CXCL12-1, sh-CXCL12-2, and control sh-NCs were synthesized from Genepharma (Shanghai, China). Lentiviruses were obtained from Genepharma (Shanghai, China). Cell transfection was performed using lipofectamine 3000 (Invitrogen, MA, USA) as the manufacturer’s protocol described. Briefly, cells were grown up to 80 % confluence and about 1 µg construct together with 1 µL lipofectamine 3000 were added into the media for 48 h. Cells were harvested for further analysis subsequently.

### Colony formation assay

Transfected MTC cells were seeded in the 12-well culture plate and grown for 10 days in the incubator to allow the colonies to form. The colonies were then fixed with 4 % PFA/sucrose and subsequently incubated 0.2 % crystal violet for 15 min. Images of stained colonies were taken and colony number was analyzed using ImageJ software.

### Cell apoptosis and cell cycle analysis

Cell apoptosis and cell cycle was evaluated by flow cytometry. Transfected cells were resuspended in binding buffer after wash with cold PBS. Suspended cells were incubated with Annexin V-FITC and propidium (PI) for 10–15 min at room temperature followed by flow cytometer analysis (Beckman, USA): AnnexinV-FITC (-) and PI (-), living cells; AnnexinV-FITC (+) and PI (-), early apoptotic cells; AnnexinV-FITC (+) and PI (+), late apoptotic cells and necrotic cells. Cells that were AnnexinV-FITC (+) /PI (-) or AnnexinV-FITC (+) /PI (+) cells were considered apoptotic cells. For cell cycle analysis, cells were fixed in 70 % ethanol at 4 °C overnight and stained with 5 µL PI for 45 min in the dark at room temperature. Then the stained cells were immediately analyzed by flow cytometry (Beckman, USA).

### Transwell migration and invasion assay

The transwell assay was carried out as described previously [26]. Transfected MTC cells were cultured in the serum-free DMEM medium on top of the filter membrane (8 μm pore) in the transwell insert. DMEM medium with 10 % FBS was placed in the lower chamber. Cells were grown for 24 h and cells growing on the lower dish were cells that migrated from top filter. They were fixed in 4 % PFA/sucrose first for 10–15 min at room temperature and 0.2 % crystal violet was added to stain the cells. To determine the invasive ability of cells, the upper chamber was pre-coated with matrigel (Corning, NY, USA) before the cells were seeded. Following 24 h of culture, cells on the bottom were cells that invaded through the filter. They were performed with similar staining process as described above.

### Scratch wound healing assay

Transfected MTC cells were seeded in 6-well plate and grown to ~ 80 % confluence. A 200 µL pipette tip was used to scratch the monolayer cells in a straight line. Cell debris were removed by PBS wash and the scratched cells were subsequently cultured in serum-free medium. Images were taken with a phase-contrast microscope at 0 and 24 h after scratch.

### RNA immunoprecipitation (RIP) assay

The cells were lysed with the lysis buffer (50 mM Tris-HCl, 150 mM NaCl, 2 mM EDTA, 1 % Triton X-100, 1 % sodium deoxycholate) supplemented with RNase inhibitors and protease inhibitor cocktail (Thermo Scientific, Waltham, MA, USA) followed by centrifuge at 17,000 g for 12 min at 4 °C. The supernatant was saved and the protein concentration was quantified with the Pierce BCA protein Assay kit (Thermo Fisher Scientific, Massachusetts, USA). Equal amount of the extracted protein was incubated with specific antibodies (anti-AGO2 or IgG, 1:500) (Abcam, MA, USA) overnight at 4 °C and then pulled down with protein G Sepharose beads (Abcam, MA, USA). The beads were washed with lysis buffer 5 times and then digested with proteinase K (Sigma-Aldrich, MO, USA) for 1.5 h. The digested solution was proceeded with RNA purification using the Trizol reagent. Quantitative RT-PCR was performed to examine the RNA yield. The primers used for analysis were listed in the qRT-PCR section.

### Dual luciferase activity assay

The wild type sequences or mutated binding sites of miR-455-5p in 3’ UTR of CXCL12 and circPVT1 were cloned into downstream of the luciferase report vector (psiCHECK2). Phusion site-directed Mutagenesis kit (Thermo Fisher Scientific, MA, USA) was used to mutate the predicted binding sites based on the manufactures’ protocol. MTC cells were seeded in 24-well culture plates first overnight and then recombinant constructs were transfected into MTC cells using lipofectamine 3000 together with miR-455-5p mimics or mimics NC. After 48 h, the cells were harvested in Reporter Lysis Buffer. The relative luciferase activity was measured by using the standard commercial Kit (Promega, WI, USA).

### Nude mice xenograft experiments

 All animal experiments have been reviewed and approved by Animal Care and Use Committee of West China Hospital of Sichuan University. The nude mice (6 weeks) were purchased from SJA Laboratory Animal Co., Ltd (Hunan, China; n = 32) and kept in laboratory conditions with 12 h/12 h light/dark cycle. MTC cells were infected with sh-NC, sh-circPVT1, mimics-NC, or miR-455-5p mimics lentiviruses for 24 h and then unilateral subaxillary subcutaneously injected into the 8-weel-old nude (5 × 10^6^ transfected MTC cells) to induce tumors. To measure the volumes of the tumors, the tumor length (L) and tumor width (W) were measured every 5 days for 30 days. The volume (V) was calculated as the following: V (mm^3^) = 0.5 × (W)^2^ × (L). At the end of experiments, the tumors were dissected out to weigh. To examine lung metastases, transfected MTC cells (5 × 10^6^) (sh-NC group, sh-circPVT1 group, mimics-NC group, and miR-455-5p mimics group) were injected through the tail vein. Lung tissues were harvested after 30 days for further H&E analysis.

### H&E staining

H&E staining was performed as the standard protocol. Briefly, lung tissues dissected from nude mice were fixed in 10 % formalin overnight at 4 °C and subsequently embedded in paraffin. Paraffin Sec. (4 μm thick) were stained with hematoxylin and eosin (H&E) as the manufacturer’s protocol described.

### RNA extraction and qRT-PCR

Total RNA was isolated from MTC tissues or cultured MTC cells by using the Trizol containing buffer (Invitrogen, Missouri, USA) as the manufacturer’s instruction described. Following RNA preparation, 1–2 µg RNA of each sample was reversely transcribed to DNA by reverse transcription kit (Thermo Fisher Scientific, MA, USA). The DNAs were amplified by PCR with PCR Master Mix Kit (Invitrogen, MA, USA). Relative expression levels of circPVT1/miR-455-5p or CXCL12 were normalized to 18 S RNA, U6 or GAPDH mRNA, respectively, as internal controls. The following primers were used:

circPVT1 forward primer: 5’-GGTTCCACCAGCGTTATTC-3’;

circPVT1 reverse primer: 5’-CAACTTCCTTTGGGTCTCC-3’;

miR-455-5p forward primer: 5’-CGGTATGTGCCTTTGGACT-3’;

miR-455-5p reverser primer: 5’-GTCGTATCCAGTGCAGGG-3’;

CXCL12 forward primer: 5’-TCAGCCTGAGCTACAGATGC-3’;

CXCL12 reverse primer: 5’-CTTTAGCTTCGGGTCAATGC-3’;

18S RNA forward primer: 5’-CAGGATTGACAGATTGATAGC-3’;

18S RNA reverse primer: 5’-GAGTCTCGTTCGT TATCGGAA-3’;

U6 forward primer:5’-CTCGCTTCGGCAGCACA-3’;

U6 reverse primer: 5’-AACGCTTCACGAATTTGCGT-3’;

GAPDH forward primer: 5’-GAGTCAACGGATTTGGTCGTT-3’;

GAPDH reverse primer: 5’-TTGATTTTGGAGGGATCTCG-3’.

### Western blot analysis

Total proteins were extracted from MTC tissues or cells by RIPA buffer (Abcam, MA, USA) supplemented with protease inhibitor cocktail. Protein concentration was quantified by BCA assay using the standard kit (Thermo Fisher Scientific, USA). Equal amounts of protein samples were loaded and separated through SDS-PAGE. Proteins in the gels were then transferred to Nitrocellulose membranes (Millipore, MA, USA). Following that, 5 % skimmed milk was used to block the membranes for half an hour at room temperature first and then primary antibodies were added to incubate at 4˚C overnight. The membranes were then washed with TBST 3 times and then incubated with corresponding secondary antibodies (KPL, USA) at room temperature for 2 h. The membranes were washed again before visualization by the ECL kit. Primary antibodies used for the study were listed as follows: anti-Snail antibody (1: 1000, Abcam, USA); anti-N-cadherin antibody (1: 1000, Abcam, USA); anti-E-cadherin antibody (1: 1000, Abcam, USA); anti-Vimentin (1: 1000, Abcam, USA); anti-CXCL12 antibody (1: 1000, Abcam, USA); anti-GAPDH (1:2000, Abcam, USA).

### Statistical analysis

All experiments were performed with at least three biological replicates, and the data were presented as Mean ± SD. All statistical analyses were performed in GraphPad Prism 7. Statistical significance was determined by unpaired Student *t* test (two groups) or one-way ANOVA (multiple groups). Patient survival curves were plotted with Kaplan-Meier method and statistic values were determined by log-rank test. The difference was considered significant if P < 0.05.

## Results

### miR-455-5p was reduced in MTC and overexpression of miR-455-5p suppressed MTC cell proliferation, migration, and invasion

**Fig. 1:**
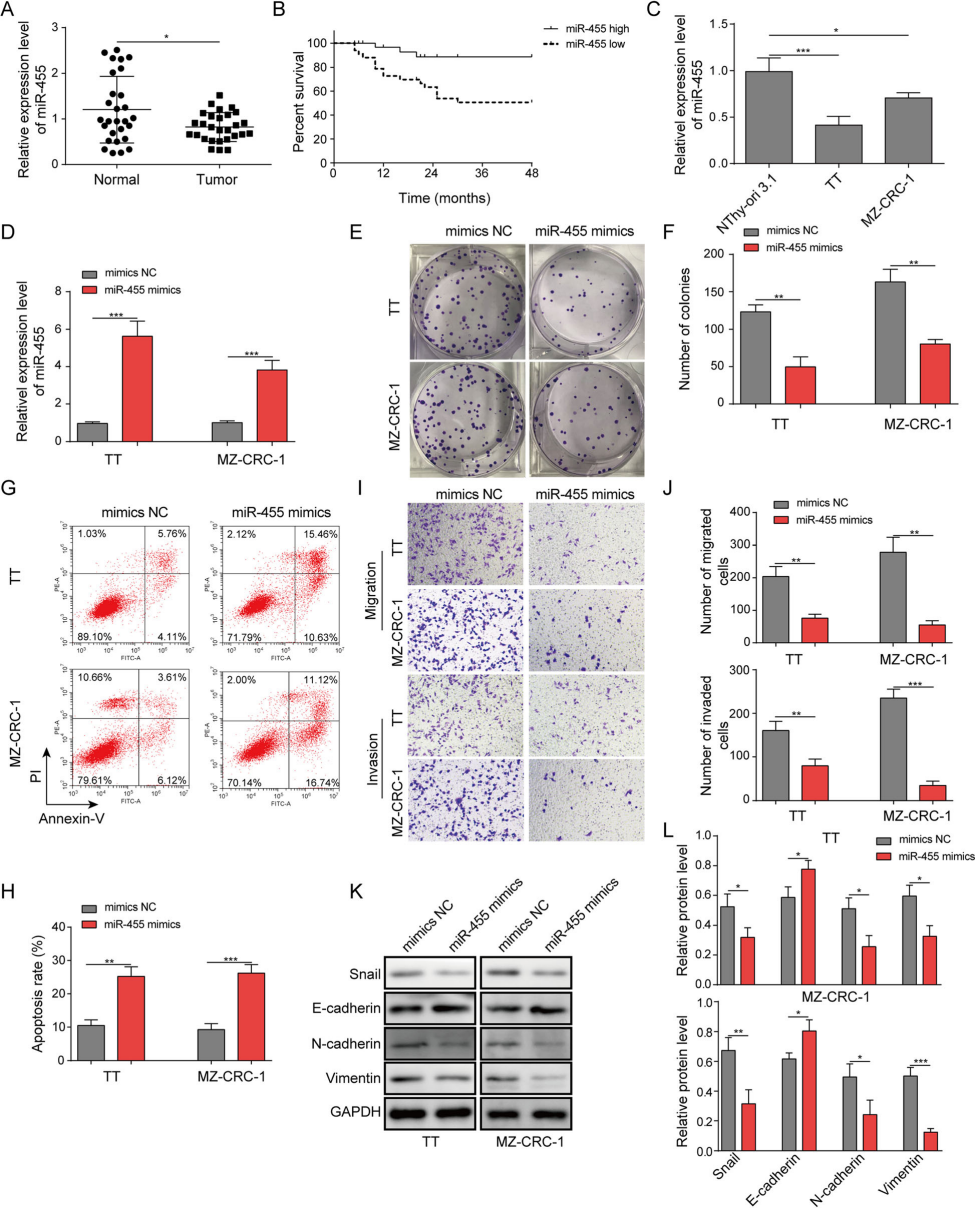
miR-455-5p was reduced in MTC and overexpression of miR-455-5p suppressed MTC cell proliferation, migration, and invasion. **a** Relative miR-455-5p levels in MTC tissues and adjacent non-tumor tissues. **b** Survival rate in patients with high miR-455-5p level and low miR-455-5p level. **c** Relative miR-455-5p levels in MTC cells. **d** Relative miR-455-5p levels in MTC cells transfected with mimics NC or miR-455-5p mimics. **e** & **f** Representative images of colonies formed in mimics NC transfected or miR-455-5p mimics transfected MTC cells. **g** Flow cytometry analysis of the number of transfected cells in G1, S, and G2 phases. **h** Flow cytometry analysis of cell apoptosis in MTC cells transfected with mimics NC or miR-455-5p mimics. **i** & **j** Transwell assay to quantify the migration and invasion abilities of transfected cells. **k** & **l** EMT-related protein levels in transfected MTC cells. * *P* < 0.05; ** *P* < 0.01; *** *P* < 0.001

To investigate the function of miR-455-5p in MTC, we first measured its level in human MTC tissues and cells. In MTC tissues from diagnosed MTC patients, we found that miR-455-5p level was significantly decreased in comparison to adjacent non-tumor normal thyroid tissues (Fig. 1a). In addition, we found the survival rate of patients that had high miR-455-5p level was consistently higher than that of patients exhibiting low miR-455-5p level (Fig. 1b). To study the molecular mechanism, we employed two of the most widely used MTC-derived cell lines (TT and MZ-CRC-1). We observed a similar change, with lower level of miR-455-5p in MTC cells than in normal thyrocytes (Fig. 1c). These results indicate that miR-455-5p is diminished in MTC. To examine the functional role of miR-455-5p in MTC, we increased the level of miR-455-5p through expression ofmiR-455-5p mimics and measured the ensuing effects on MTC development. As expected, we confirmed that miR-455-5p level was robustly increased in miR-455-5p mimics transfected cells in comparison to mimics NC transfected cells (Fig. 1d). With flow cytometry, we observed significant more cells in G1 phase following transfection of miR-455-5p mimics compared to mimics NC transfection (Fig. 1e). Using colony formation assay, we found that miR-455-5p mimics greatly decreased the number of colonies formed (Fig. 1f&g). Further, the flow cytometry results showed that miR-455-5p mimics increased the percentage of apoptotic cells (Fig. 1h&i). We then employed transwell assay to examine the migration and invasion abilities of MTC cells. We observed that MTC cells transfected with miR-455-5p mimics had lower numbers of migrated and invasive cells than cells transfected with mimics NC (Fig. 1j&k). To examine the EMT process, a key process during the cancer development, we performed Western blotting to determine the protein levels of EMT-related proteins. We found miR-455-5p mimics increased E-cadherin protein level but diminished protein levels of N-cadherin, Snail, and Vimentin (Fig. 1l&m), suggesting that miR-455-5p mimics represses EMT process of cancer cells. With scratch wound healing assay, we found that miR-455-5p mimics significantly diminished the migration distance of MTC cells (Fig. 1n&o). Taken together, these results show that miR-455-5p is diminished in MTC cells and that increasing miR-455-5p level inhibits MTC cell proliferation, migration, and invasion.

### miR-455-5p suppressed CXCL12 signaling via targeting CXCL12

**Fig. 2:**
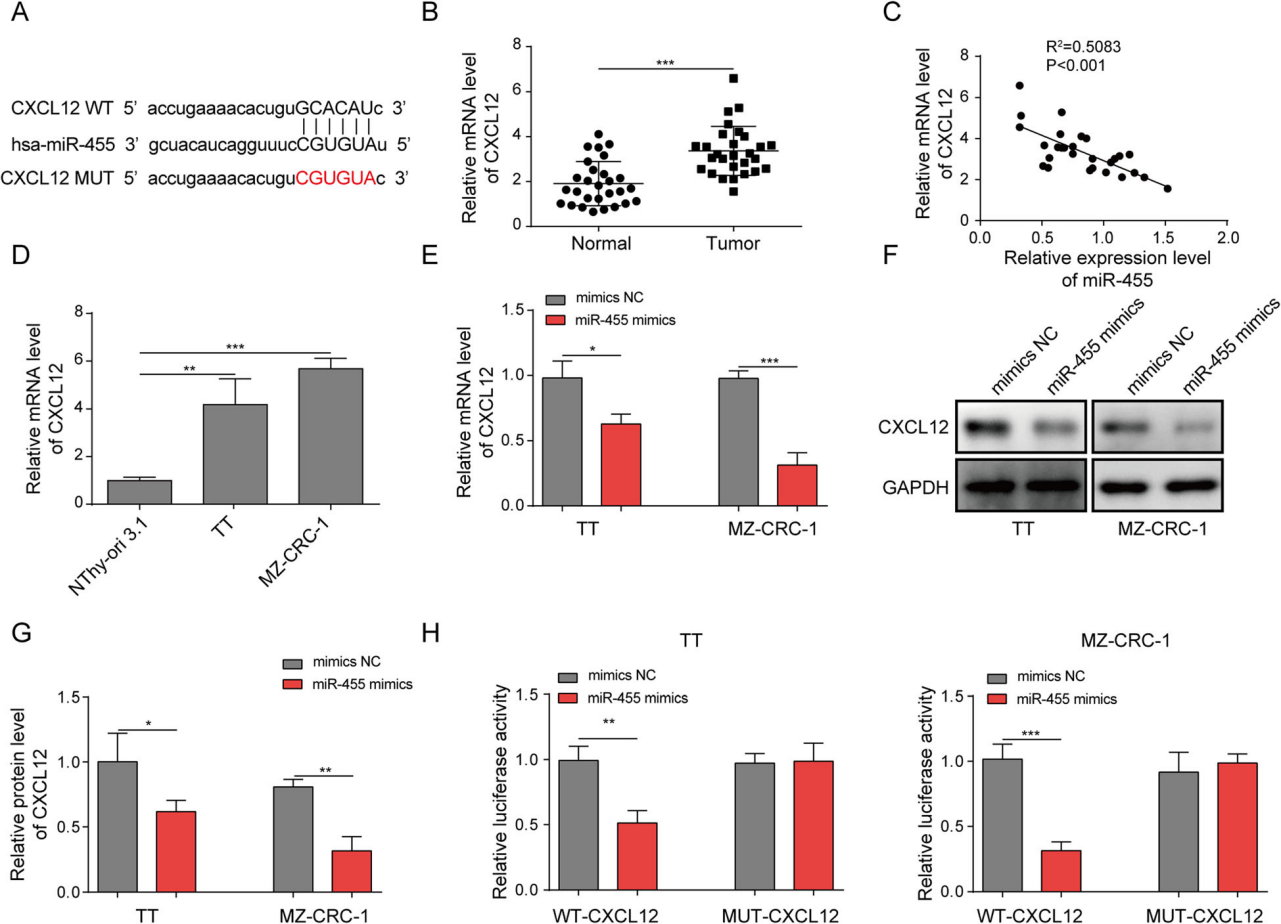
miR-455-5p suppressed CXCL12 signaling via targeting CXCL12. **a** Complementary binding sites between CXCL12 3’ UTR and miR-455-5p. **b** Relative CXCL12 mRNA levels in MTC tissues. **c** Correlation between miR-455-5p level and CXCL12 mRNA level in MTC tissues. **d** Relative CXCL12 mRNA level in MTC cells. **e** Relative CXCL12 mRNA level in mimics NC transfected or miR-455-5p mimics transfected MTC cells. **f **& **g** Relative CXCL12 protein level in mimics NC transfected or miR-455-5p mimics transfected MTC cells. **h** Relative luciferase activity in transfected MTC cells. *P < 0.05; ** *P* < 0.01; *** *P* < 0.001

miRNAs usually exert their functions by targeting downstream mRNAs. Through bioinformatic analysis (Starbase, http://starbase.sysu.edu.cn/index.php), we identified some complementary binding sites between miR-455-5p and CXCL12 mRNA (Fig. 2a), suggesting that they two may bind to each other. Indeed, we found that CXCL12 mRNA level was up-regulated in MTC tissues (Fig. 2b), which is opposite to miR-455-5p change. We also detected a significant inverse correlation between miR-455-5p level and CXCL12 mRNA level in MTC specimens (Fig. 2c). CXCL12 mRNA level was elevated as well in MTC cells (Fig. 2d). Cells transfected with miR-455-5p mimics had significantly lower levels of CXCL12 mRNA and protein (Fig. 2e-g). Moreover, with dual luciferase activity assay, we observed that miR-455-5p mimics greatly decreased the relative luciferase activity of WT-CXCL12 but not MUT-CXCL12 wherein the predicted binding sites with miR-455-5p were mutated (Fig. 2h). Therefore, we conclude that miR-455-5p directly targets CXCL12 and negatively regulates its expression.

### Inhibition of CXCL12/CXCR4 signaling blocked the effects of miR-455-5p inhibitor on MTC cell proliferation, migration, and invasion

**Fig. 3:**
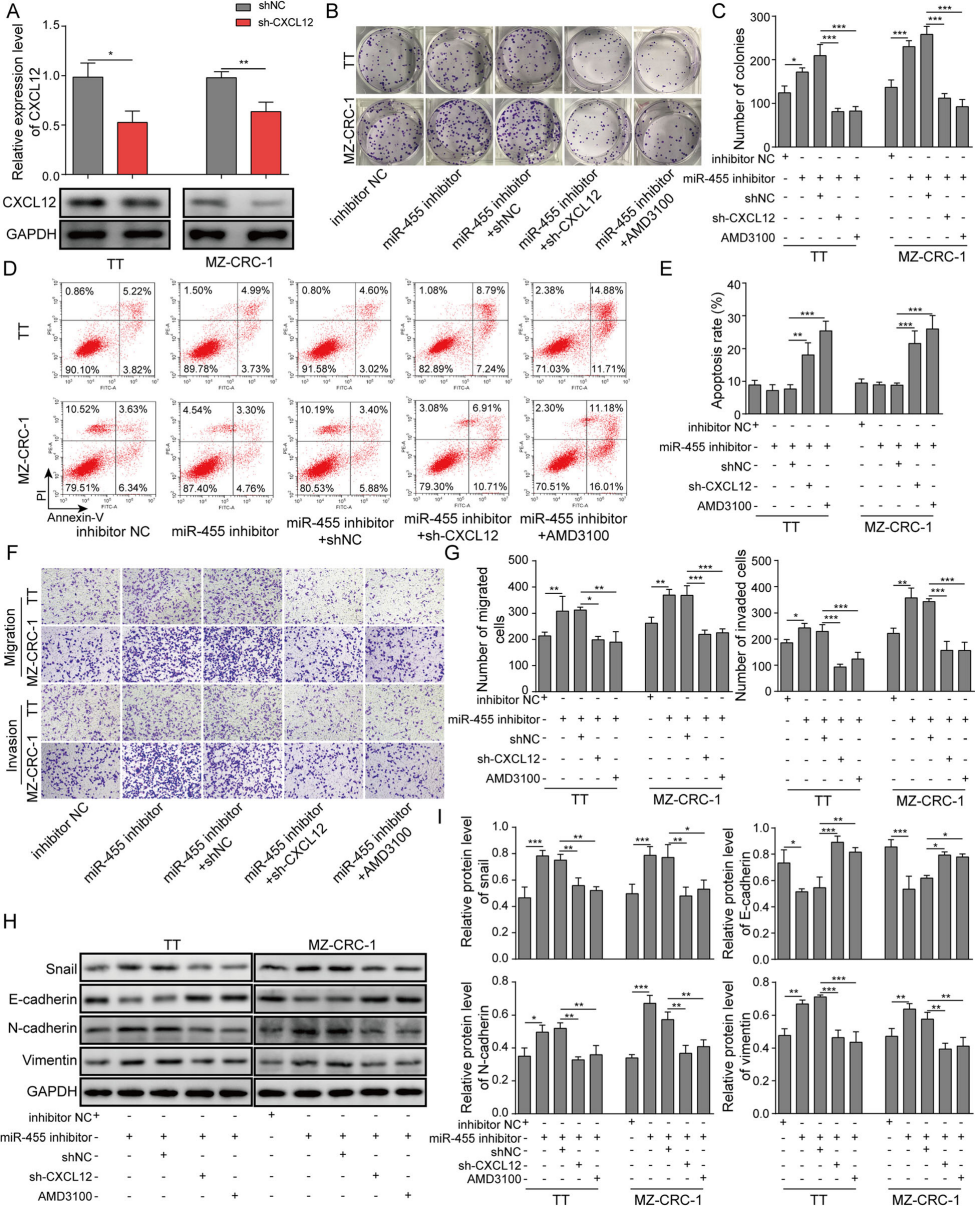
Inhibition of CXCL12/CXCR4 signaling blocked the effects of miR-455-5p inhibitor on MTC cell proliferation, migration, and invasion. **a** Relative CXCL12 mRNA and protein levels in MTC cells transfected shNC or shCXCL12. **b** & **c** Representative images of colonies formed in MTC cells transfected miR-455-5p mimics or shCXCL12 or treated with CXCR4 antagonist AMD3100. **d** Flow cytometry analysis of the number of transfected cells in G1, S, and G2 phases. **e** Flow cytometry analysis of cell apoptosis in MTC cells transfected miR-455-5p mimics or shCXCL12 or treated with CXCR4 antagonist AMD3100. **f** & **g** Transwell assay to analyze the migration and invasion abilities of transfected cells. **h** & **i** EMT-related protein levels in MTC cells transfected miR-455-5p mimics or shCXCL12 or treated with CXCR4 antagonist AMD3100. * *P* < 0.05; ** *P* < 0.01; *** *P* < 0.001

We then examined the function of miR-455-5p/CXCL12 interaction in MTC. As expected, cells transfected with shCXCL12 had lower levels of CXCL12 mRNA and protein (Fig. 3a). In contrast to the effects of miR-455-5p mimics, miR-455-5p inhibitor remarkably increased the number of colonies formed and cells in the phases of S and G2, but decreased the percentage of apoptotic cells (Fig. 3b-e). However, the increases in colony number and the number of cells in S phases, plus the reduction in percentage of apoptotic cells, were reversed when cells were co-transfected with miR-455-5p inhibitor and shCXCL12 or when miR-455-5p inhibitor-transfected MTC cells were treated with AMD3100, the CXCR4 antagonist (Fig. 3b-f). Similarly, miR-455-5p inhibitor greatly enhanced the migration and invasion abilities of MTC cells while sh-CXCL12 and AMD3100 blocked that enhancement (Fig. 3g&h). Regarding the EMT process, our Western blotting results indicated that miR-455-5p inhibitor promoted EMT process by diminishing E-cadherin protein level and up-regulating Snail, N-cadherin, and Vimentin levels (Fig. 3i&j). This promotion was reversed by shCXCL12 and AMD3100 (Fig. 3i&j). In addition, we observed that cells transfected with miR-455-5p inhibitor exhibited elongated mesenchymal shapes, and the effect of miR-455-5p inhibitor on cell morphology was blocked by shCXCL12 and AMD3100 (Fig. S1A). Together, these data prove that miR-455-5p regulates MTC development via CXCL12/CXCR4 signaling.

### circPVT1 was elevated in MTC specimens and activated CXCL12 signaling via miR-455-5p

**Fig. 4:**
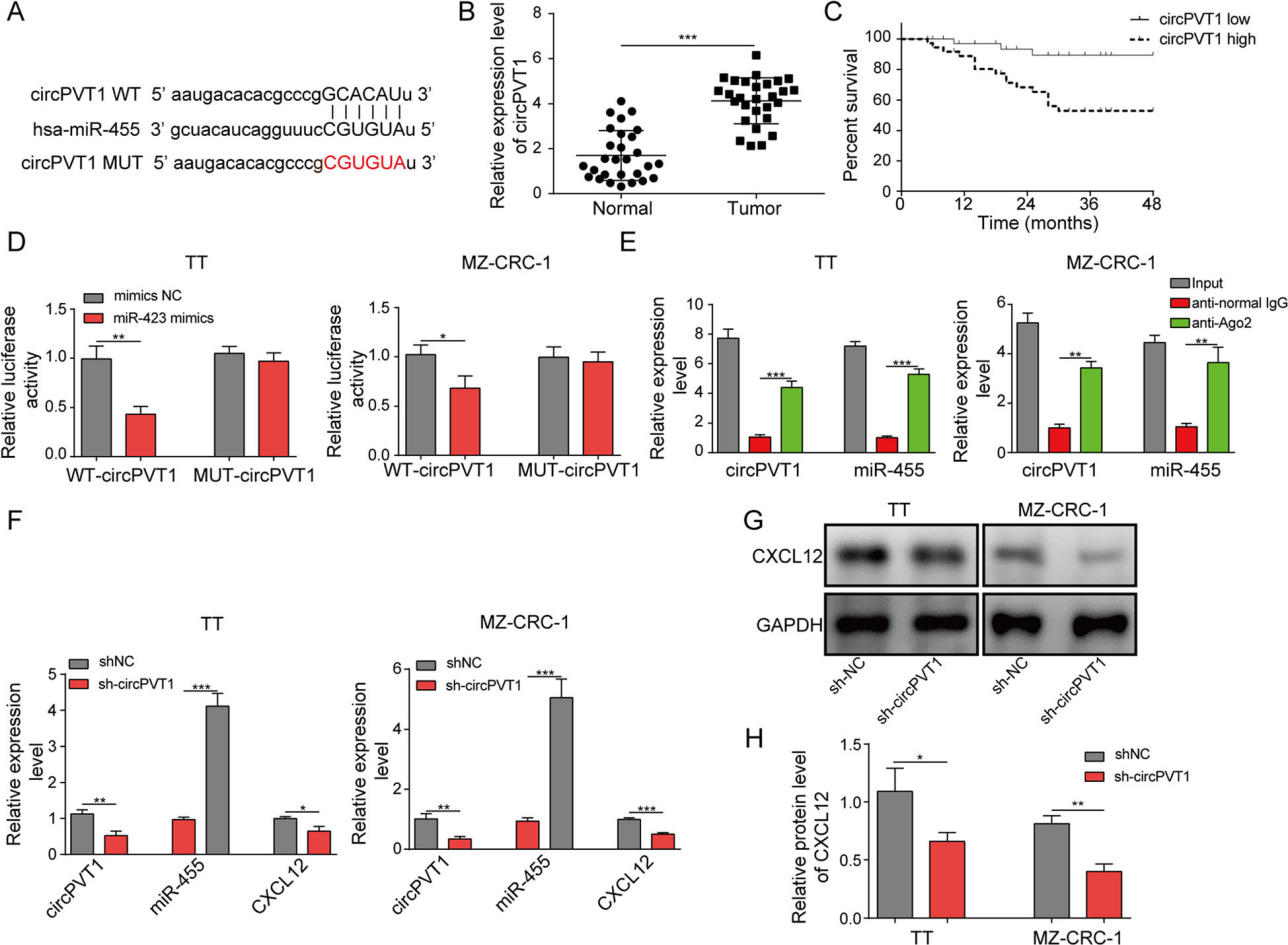
circPVT1 activated CXCL12 signaling via binding miR-455-5p. **a** Complementary binding sites between circPVT1 and miR-455-5p. **b** Relative circPVT1 levels in MTC tissues. **c** Survival rate of MTC patients with high circPVT1 level or low circPVT1 level. **d** Relative luciferase activity in transfected MTC cells. **e** Relative circPVT1 and miR-455-5p levels pulled down by Ago2 antibodies. **f** Relative circPVT1, miR-455-5p, and CXCL12 levels in MTC cells transfected with shNC or shcircPVT1. **g **& **h** Relative CXCL12 protein level in MTC cells transfected with shNC or shcircPVT1. *P < 0.05; ** *P* < 0.01; *** *P* < 0.001

Next, we went to study the upstream regulator of miR-455-5p in MTC. We again performed bioinformatic analysis through Starbase and detected complementary binding sites between circPVT1 and miR-455-5p (Fig. 4a). To test whether circPVT1 regulate miR-455-5p in MTC, we first measured its level in MTC. We found circPVT1 was up-regulated in human MTC tumor specimens (Fig. 4b). Moreover, patents with high circPVT1 level had lower survival rates than patients with low circPVT1 level (Fig. 4c). We then directly validated the interaction through dual luciferase activity assay. The luciferase activity of WT-circPVT1 was remarkably diminished by miR-455-5p mimics while the activity of MUT-circPVT1 harboring the mutated binding sites with miR-455-5p was not affected by miR-455-5p mimics (Fig. 4d). In addition, we showed that immunoprecipitation with specific Ago-2 antibody significantly enriched circPVT1 and miR-455-5p (Fig. 4e). These data demonstrate that circPVT1 directly interacts with miR-455-5p. We then examined how circPVT1 regulated miR-455-5p and CXCL12 signaling. shcircPVT1 drastically diminished circPVT1 level in transfected MTC cells (Fig. 4f). In shcircPVT1-transfected cells, we observed an increased miR-455-5p expression and a reduced level of CXCL12 mRNA and protein compared to shNC-transfected cells (Fig. 4f-h). Together, our data show that circPVT1 acts a miR-455-5p sponge to activate CXCL12 signaling.

### Knockdown of circPVT1 suppressed MTC cell proliferation, migration, and invasion

**Fig. 5:**
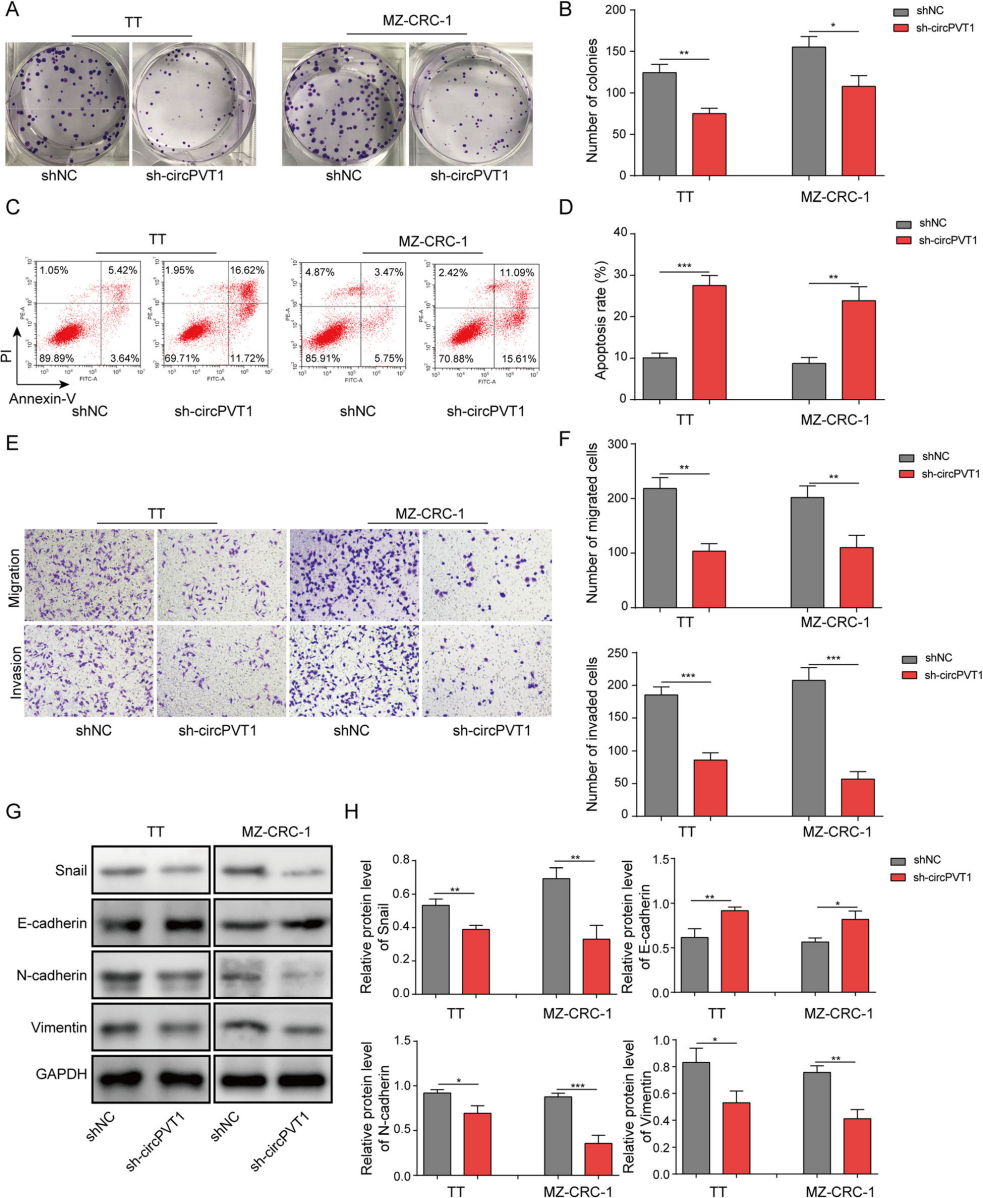
Knockdown of circPVT1 suppressed MTC cell proliferation, migration, and invasion. **a** & **b** The number of colonies formed in shNC transfected or shcircPVT1 transfected MTC cells. **c** Flow cytometry analysis of the number of transfected cells in G1, S, and G2 phases. **d** Flow cytometry analysis of cell apoptosis in MTC cells transfected with shNC or shcircPVT1. **e** & **f** Transwell assay and scratch wound healing assay to analyze migration and invasion abilities of transfected cells. **g** & **h** EMT-related protein levels in MTC cells transfected with shNC or shcircPVT1. * *P* < 0.05; ** *P* < 0.01; *** *P* < 0.001

Next, we tested the function of circPVT1 in MTC. Knockdown of circPVT1 increased the number of G1 cells (Fig. 5a). With colony formation assay, we found knockdown of circPVT1 with sh-circPVT1 greatly decreased the number of colonies formed (Fig. 5b&c). In contrast, as shown in Fig. 5d&e, knockdown of circPVT1 significantly increased the percentage of apoptotic cells. Using transwell assay, we showed that cells transfected with sh-circPVT1 had reduced migration and invasion abilities compared with shNC transfected cells (Fig. 5f&g). Similarly, with scratch wound healing approach, we found that knockdown of circPVT1 significantly diminished the migration distance of transfected cells (Fig. 5h&i). Western blotting data indicated that knockdown of circPVT1 increased E-cadherin level but decreased levels of Snail, N-cadherin, and Vimentin (Fig. 5j&k). Moreover, cells with overexpression of circPVT1 exhibited elongated mesenchymal shapes (Fig. S1B). These results provide evidence that knockdown of circPVT1 inhibits MTC cell proliferation, migration, invasion and EMT process.

### circPVT1 regulated MTC development via miR-455-5p/CXCL12 axis

**Fig. 6:**
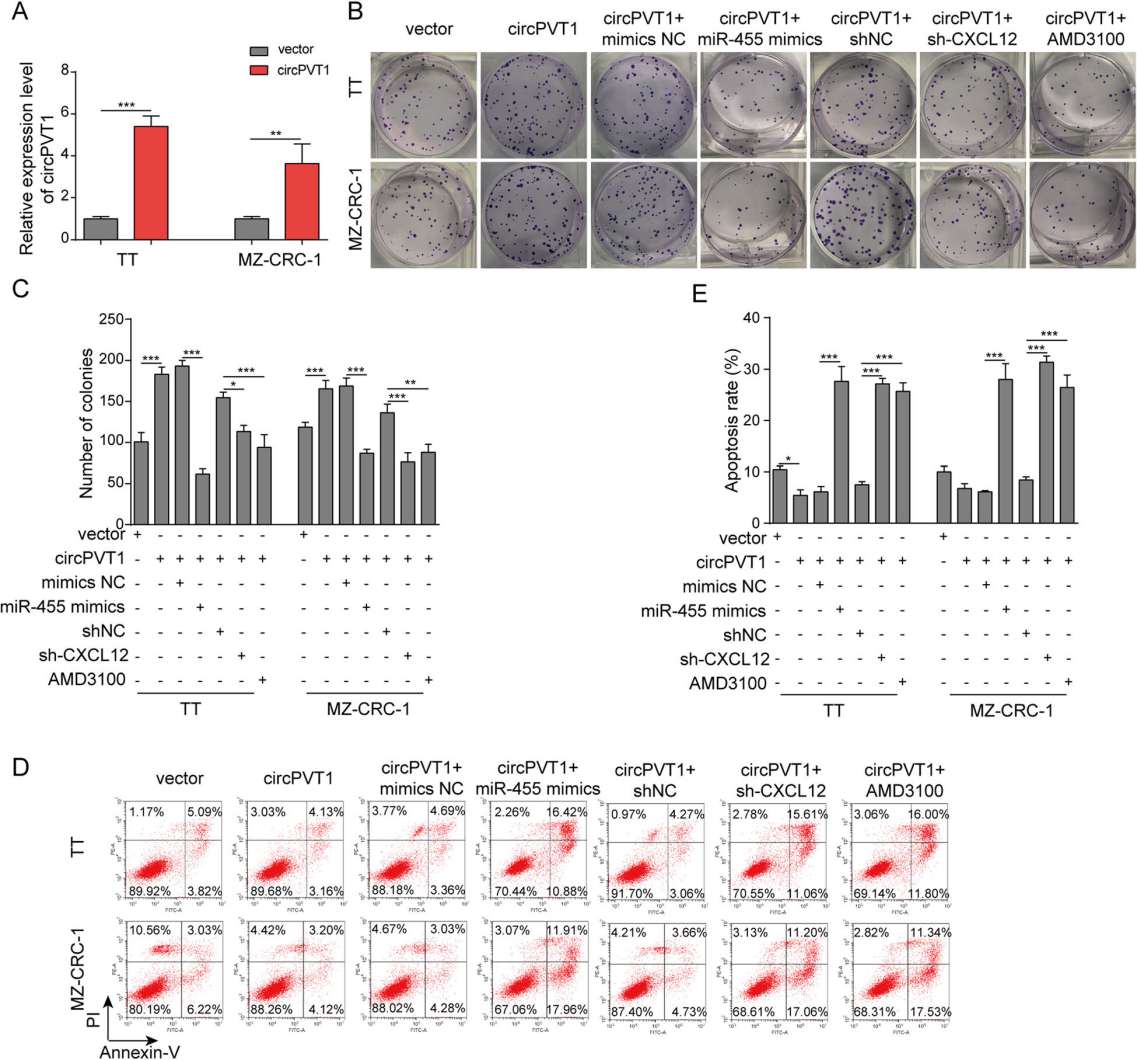
circPVT1 regulated MTC cells proliferation and apoptosis via miR-455-5p/CXCL12 axis. **a** Relative circPVT1 level in MTC cells transfected circPVT1 overexpressing vector. **b** & **c** The number of colonies formed in MTC cells transfected with circPVT1 overexpressing vector or miR-455-5p mimics or treated with CXCR4 antagonist AMD3100. **d** Flow cytometry analysis of the number of transfected cells in G1, S, and G2 phases. **e** Flow cytometry analysis of cell apoptosis in MTC cells transfected with circPVT1 overexpressing vector or miR-455-5p mimics or treated with CXCR4 antagonist AMD3100. * *P* < 0.05; ** *P* < 0.01; *** *P* < 0.001

**Fig. 7:**
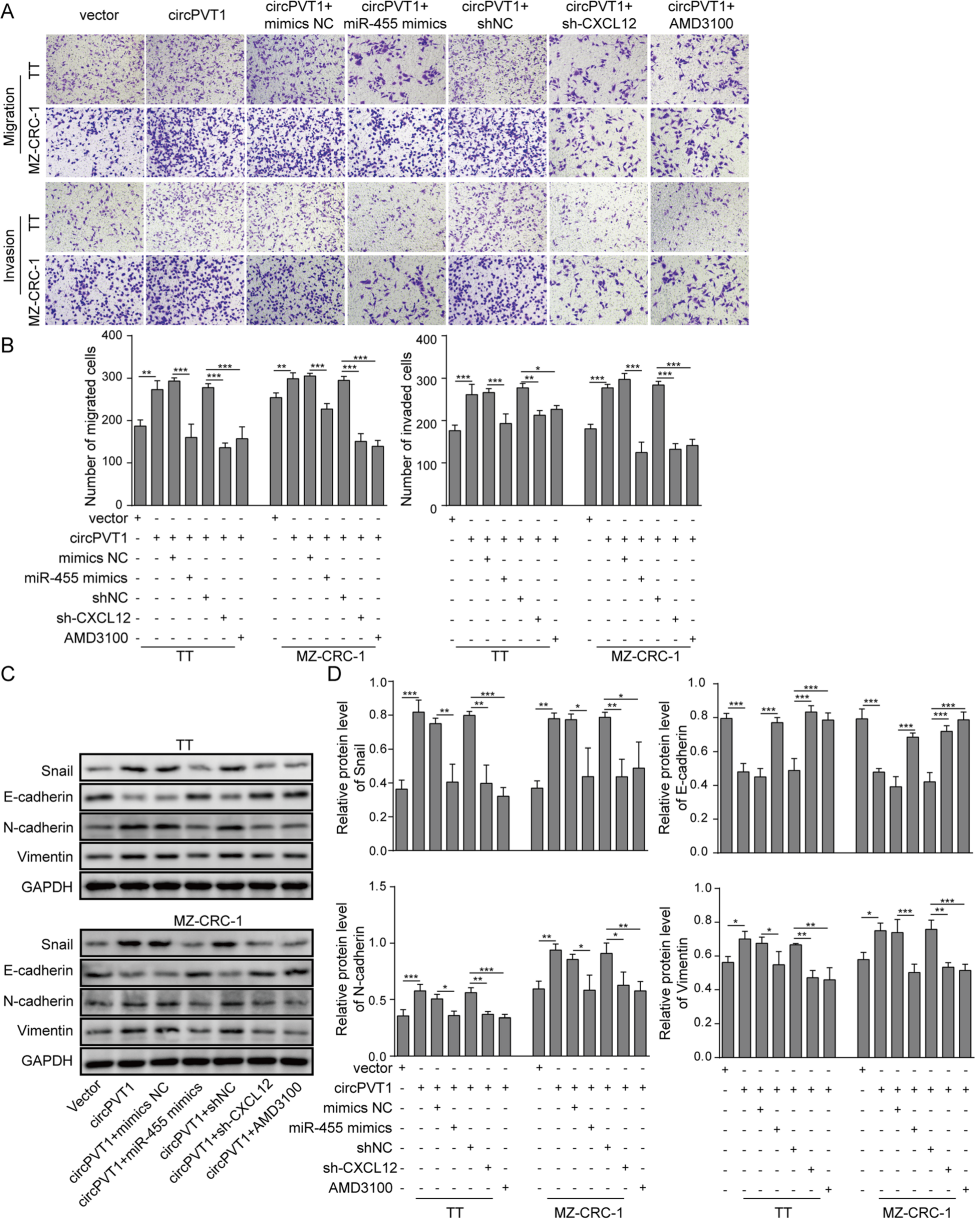
circPVT1 regulated MTC cells migration, invasion and EMT process via miR-455-5p/CXCL12 axis. **a **& **b** Transwell assay to analyze migration and invasion abilities of transfected cells. **c **& **d** EMT-related protein levels in MTC cells transfected with circPVT1 overexpressing vector or miR-455-5p mimics or treated with CXCR4 antagonist AMD3100. * *P* < 0.05; ** *P* < 0.01; *** *P* < 0.001

We then investigated the mechanisms underlying the role of circPVT1 in MTC. Transfection of MTC cells with circPVT1 tremendously increased circPVT1 in cells (Fig. 6a). transfection of cells with circPVT1 diminished the number of G1 cells but raised the number of S and G2 cells while co-transfection of miR-455-5p mimics or shCXCL12, or treatment with AMD3100 reversed those changes (Fig. 6b). Moreover, with colony formation assay, as expected, overexpression of circPVT1 significantly increased the number of colonies formed (Fig. 6c&d). However, this increase was reversed when cells were co-transfected with miR-455-5p mimics or shCXCL12, or treated with CXCR4 antagonist AMD3100 (Fig. 6b&c). As shown in Fig. 6e&f, overexpression of circPVT1 reduced the percentage of apoptotic cells while co-expression of miR-455-5p or shCXCL12, or treatment with AMD3100 recovered the reduction. Similarly, with transwell assay, we showed that overexpression of circPVT1 increased the numbers of migration and invasion cells while co-expression with miR-455-5p or shCXCL12 or AMD3100 treatment inhibited the increase (Fig. 7a&b). Regarding EMT process, transfection of circPVT1promoted the EMT by decreasing E-cadherin and increasing Snail, N-cadherin, and Vimentin (Fig. 7c&d). Again, the promotion induced by circPVT1 was blocked by miR-455-5p mimics, shCXCL12, or AMD3100 (Fig. 7c&d). Furthermore, the effect of circPVT1 overexpression on cell morphology was reversed as well by miR-455-5p mimics, shCXCL12, or AMD3100 (Fig. S1B). Taken together, these results show that circPVT1 promotes MTC cell proliferation, migration, and invasion via miR-455-5p/CXCL12/CXCR4 pathway.

### Knockdown of circPVT1 and overexpression of miR-455-5p repressed MTC tumor growth and lung metastasis *in vivo*

**Fig. 8:**
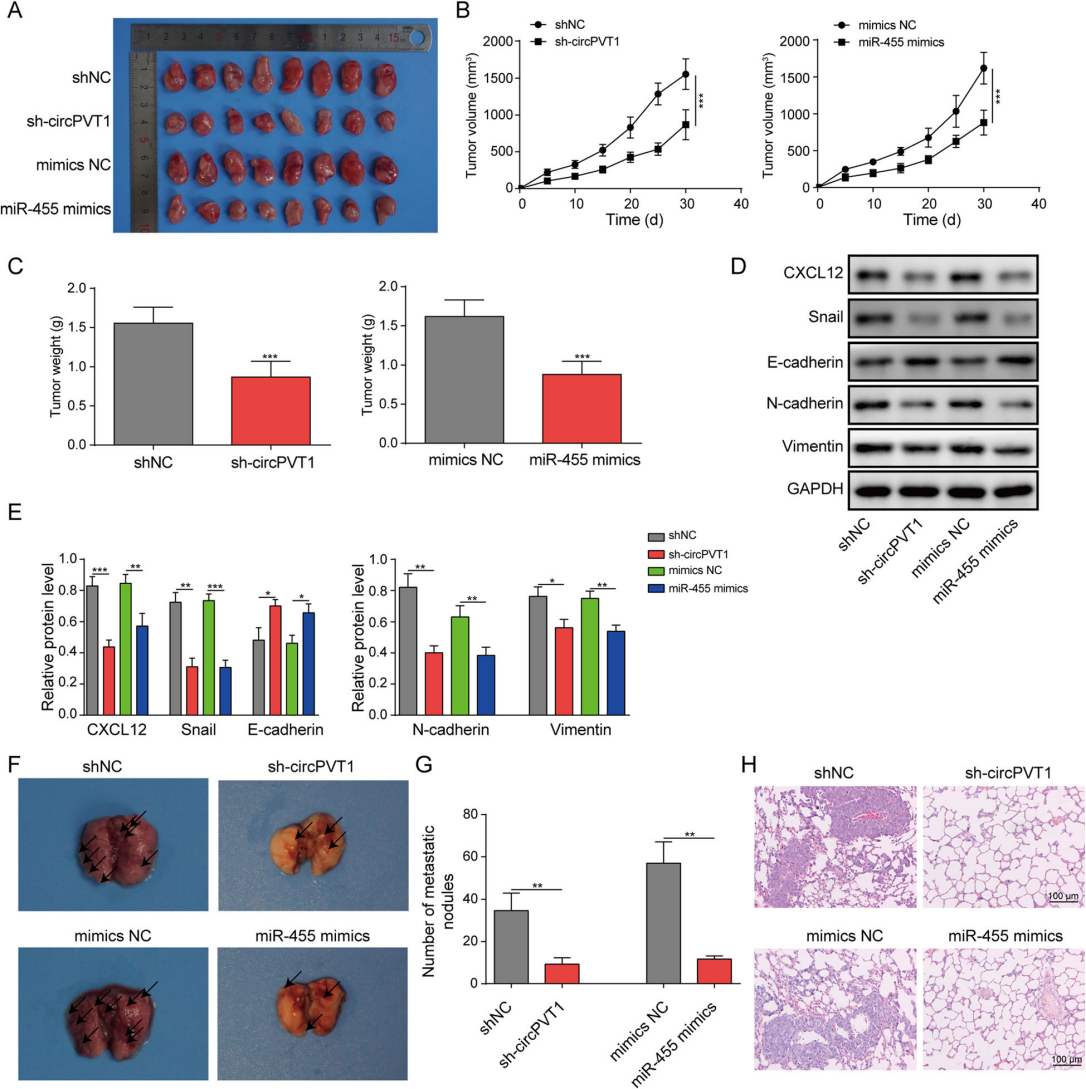
Knockdown of circPVT1 and overexpression of miR-455-5p repressed MTC tumor growth and lung metastasis *in vivo.* **a** Representative tumor images in each group of mice. **b** The tumor volume in each group of mice with time. **c** The tumor weight in each group of mice. **d **& **e** CXCL12 and EMT-related protein levels in the tumors from each group of mice. **f** Representative images of the lung tissues from each group of mice. **g** Relative numbers of tumor nodules in the lung from each group of mice. **h** Representative H&E staining images of the lung from each group of mice. * *P* < 0.05; ** *P* < 0.01; *** *P* < 0.001

In the end, we evaluated the function of circPVT1 and miR-455-5p in MTC progression *in vivo* by using the nude mouse xenograft model. We infected MTC cells with-sh-circPVT1 or miR-455-5p mimics and delivered those MTC cells into the nude mice. As shown in Fig. 8a&b, the volumes of tumors progressively increased with time in the control group mice bearing the shNC-transfected or mimics NC-transfected MTC cells. Nevertheless, the tumors from mice implanted with shcircPVT1-transfected MTC cells or miR-455-5p mimics-transfected MTC cells were significantly and consistently smaller (Fig. 8a&b). The tumors in shcircPVT1 group and miR-455-5p mimics group weighed less as well compared to the tumors in the control groups (Fig. 8c). In the end, we measure some protein levels in the tumor tissues from each group. We found that both knockdown of circPVT1 and overexpression of miR-455-5p decreased CXCL12 protein level in the tumors (Fig. 8d&e). In addition, they suppressed the EMT process as both shcircPVT1 and miR-455-5p mimics up-regulated E-cadherin level but down-regulating Snail, N-cadherin, and Vimentin (Fig. 8d&e). These results indicate that knockdown of circPVT1 or overexpression of miR-455-5p restrains MTC tumor growth *in vivo*. Additionally, to examine the lung metastasis, we injected the transfected MTC cells through the tail vein to the nude mice and dissected out the lung tissues after 30 days. With the H&E staining, we found that the number of tumor nodules in the lung from the shcircPVT1 group or from the miR-455-5p mimics group was significantly smaller than from control groups (Fig. 8f-h). Therefore, knockdown of circPVT1 or overexpression of miR-455-5p inhibits MTC lung metastasis in animals.

## Discussion

MTC is an uncommon type of thyroid cancer deriving from parafollicular C cells [1, 2]. Despite tremendous advances in research, the mechanisms that contribute to MTC are still not clear. Previous work suggested that miR-455-5p was dysregulated in MTC, but the exact functional role of miR-455-5p in MTC remains elusive [14]. Here, we fully elucidated the function of miR-455-5p in MTC. We found that miR-455-5p was reduced in MTC tissues and cells. Rescue of miR-455-5p level suppressed MTC cell proliferation, migration, and invasion. Significantly, we showed that circPVT1 directly bound to miR-455-5p while CXCL12/CXCR4 signaling was the downstream target of miR-455-5p. miR-455-5p regulated MTC development through targeting CXCL12 while circPVT1 acted as a miR-455-5p sponge. Knockdown of circPVT1 or overexpression of miR-455-5p inhibited MTC tumor growth and metastasis *in vivo*. These results provide strong evidence that circPVT1/miR-455-5p modulates MTC via CXCL12/CXCR4 signaling. Notably, we used adjacent non-tumor medullary thyroid tissues and the normal human thyrocyte cell line as control. It might be better to include the benign c-cell tissues and a normal neuroendocrine cell line.

Since their first discoveries, many circRNAs have been implicated in diseases particularly in cancers [15, 16]. Some function as oncogenes while some act as tumor suppressors. Some circRNAs have complicated roles depending on types of cancers [17]. In thyroid cancers, aberrant expressions of circRNAs have been reported as well [27]. For example, circBACH2 contributes to papillary thyroid carcinoma [28]. Emerging evidence indicates that circPVT1 is an oncogenic circRNA. It promotes the development and metastasis of colorectal cancer [29]. Also, it accelerates proliferation, migration, and invasion of non-small-cell lung cancer cells [19, 30, 31]. However, in MTC, the role of circPVT1 is not clear. Here, we showed that circPVT1 contributed to MTC development. Knockdown of circPVT1 suppressed MTC cell proliferation, migration and invasion, and inhibited MTC tumor growth and lung metastasis *in vivo*. Our study, together with previous studies [18, 19, 29–31], supports the model that circPVT1 is a general oncogene non-coding RNA. Due to the circular structure, circRNAs are usually very stable and thus can be used as diagnosis markers of diseases. Here, we showed that high level of circPVT1 correlated well with the poor prognosis of MTC. Therefore, elevation of circPVT1 could potentially serve as an early diagnosis marker for MTC.

Many circRNAs regulate gene expression by acting as competing endogenous RNAs (ceRNAs) to sponge miRNAs, although some other mechanisms exist as well [32, 33]. It is well known that miRNAs are a very important class of regulators for diseases like cancers [9, 34]. A huge body of literature show that enormous miRNAs are involved in cancer development and progression [35, 36]. miR-455-5p is usually considered as a tumor suppressor since may studies show that its level is down-regulated in cancer tissues. For instance, in colorectal cancer, expression of miR-455-5p is sharply reduced and overexpression of miR-455-5p greatly inhibited colorectal cancer cell proliferation and migration [11]. In breast cancer, miR-455-5p has similar tumor suppressor effect [37]. In MTC, we observed that miR-455-5p was remarkably diminished and that overexpression of miR-455-5p repressed MTC cell growth and metastasis both *in vitro* and *in vivo*. Mechanistically, we showed that miR-455-5p was the downstream target of circPVT1. circPVT1 sponged miR-455-5p to promote MTC growth and metastasis. Previous studies have shown that circPVT1 has multiple targets, such as miR-26b, miR-149, and miR-203a [38–40]. For instance, it has been shown that circPVT1 promotes the development and progression of non-small cell lung cancer by sponging miR-125b [30]. Future research is required to examine the role of those interactions in MTC. In addition, whether circPVT1/miR-455-5p interaction is involved in other diseases or cancers remains further investigation.

miRNAs exert their functions by regulating gene expressions through targeting mRNAs [41]. In the study, we found that miR-455-5p bound CXCL12 3’ UTR and negatively regulated its expression. Chemokines are key players in the inflammation, as well as tumors [42, 43]. Chemokine CXCL12 has angiogenic properties and greatly contributes to the outgrowth and metastasis of CXCR4-expressiong cancers [21, 44]. Numerous studies have shown that both CXCL12 and its receptor CXCR4 are up-regulated in various cancers [45–47]. These up-regulations could stimulate diverse downstream pathways including NF-κB signaling and AKT/MAPK pathway, thereby promoting cancer cell proliferation and invasion [46]. In addition, enhanced CXCL12/CXCR4 signaling can induce cells to secrete growth factors and cytokines to create a beneficial microenvironment for tumor growth [48]. As a result, CXCL12/CXCR4 axis plays a crucial role in cancer and could serve as a target for cancer therapy [49]. Consistent with previous studies, our work also indicates a key role of CXCL12/CXCR4 signaling in MTC. By targeting CXCL12, circPVT1/miR-455-5p strongly affects MTC progression. Many other targets of miR-455-5p have been reported, such as galectin-9 and PIK3R1 and these interactions have been implicated in cancer development [11, 50]. It will be interesting to examine whether those signaling pathways are involved in MTC as well. On the other hand, it will be interesting to test whether miR-455-5p/CXCL12 interaction is implicated in other diseases or cancers in the future.

## Conclusions

In summary, we demonstrate that circPVT1 promotes MTC growth and metastasis by targeting miR-455-5p to activate CXCL12/CXCR4 signaling pathway. circPVT1 and miR-455-5p may be key regulators in MTC and play a crucial role in MTC development and progression. In the future, more clinical data and *in vivo* experiments on circPVT1 and miR-455-5p are needed to verify the results at the *in vitro* cell level. Treatments targeting these molecular might be useful to treatment MTC.

### Supplementary Information

**Additional file 1: Figure S1.** circPVT1/miR-455-5p regulated EMT viaCXCL12.(A&B) Morphologies of transfected cells.

## Data Availability

All data generated or analyzed during this study are included in this published article.

## Abbreviations

MTC: Medullary thyroidcancer
MEN2: Multiple endocrineneoplasia 2
RET: Rearranged duringTransfection
miRNAs: MicroRNAs
circRNAs: Circular RNAs
DMEM: Dulbecco’sModified Eagle Medium
P/S: Penicillin-streptomycin
PI: Propidium
RIP: RNAImmunoprecipitation
L: Length
W: Width
V: Thevolume
H&E: Hematoxylin and eosin
ceRNAs: Competingendogenous RNAs
