# Correction: circPVT1 regulates medullary thyroid cancer growth and metastasis by targeting miR-455-5p to activate CXCL12/CXCR4 signaling

**DOI:** 10.1186/s13046-023-02880-1

**Published:** 2023-11-14

**Authors:** Xun Zheng, Shu Rui, Xiao-Fei Wang, Xiu-He Zou, Yan-Ping Gong, Zhi-Hui Li

**Affiliations:** Department of Thyroid and Parathyroid Surgery Center, West China Hospital, Sichuan University, No. 37 Guo Xue Xiang, 610041 Chengdu, Sichuan China

**Correction: *J Exp Clin Cancer Res*** **40, 157 (2021)**

10.1186/s13046-021-01964-0

Following the publication of the original article [1], authors would like to correct he typographical error in the cell line under Materials and Methods, Cell Culture section.

The correct sentence should read as:

Two human MTC cell lines (TT, MZ-CRC-1), and one normal human thyrocyte cell line (NThy-ori3.1) were used for the study as TT and MZ-CRC-1 are the two of most widely used MTC-derived cell lines [23–25].

Furthermore, the figure legends for figure 1, 3, 5 and 6 are in the wrong order. The right order for these figure legends are given below:

**Fig. 1** miR-455-5p was reduced in MTC and overexpression of miR-455-5p suppressed MTC cell proliferation, migration, and invasion. **a** Relative miR-455-5p levels in MTC tissues and adjacent non-tumor tissues. **b** Survival rate in patients with high miR-455-5p level and low miR-455-5p level. **c** Relative miR-455-5p levels in MTC cells. **d** Relative miR-455-5p levels in MTC cells transfected with mimics NC or miR-455-5p mimics. **e** & **f** Representative images of colonies formed in mimics NC transfected or miR-455-5p mimics transfected MTC cells. **g** Flow cytometry analysis of the number of transfected cells in G1, S, and G2 phases. **h** Flow cytometry analysis of cell apoptosis in MTC cells transfected with mimics NC or miR-455-5p mimics. **i** & **j** Transwell assay to quantify the migration and invasion abilities of transfected cells. k & l EMT-related protein levels in transfected MTC cells. *P < 0.05; ** *P* < 0.01;*** *P* < 0.001

**Fig. 3** Inhibition of CXCL12/CXCR4 signaling blocked the effects of miR-455-5p inhibitor on MTC cell proliferation, migration, and invasion. **a** Relative CXCL12 mRNA and protein levels in MTC cells transfected shNC or shCXCL12. **b** & **c** Representative images of colonies formed in MTC cells transfected miR-455-5p mimics or shCXCL12 or treated with CXCR4 antagonist AMD3100. **d** Flow cytometry analysis of the number of transfected cells in G1, S, and G2 phases. **e** Flow cytometry analysis of cell apoptosis in MTC cells transfected miR-455-5p mimics or shCXCL12 or treated with CXCR4 antagonist AMD3100. **f** & **g** Transwell assay to analyze the migration and invasion abilities of transfected cells. **h** & **i** EMT-related protein levels in MTC cells transfected miR-455-5p mimics or shCXCL12 or treated with CXCR4 antagonist AMD3100. *P < 0.05; ** *P* < 0.01; *** *P* < 0.001

**Fig. 5** Knockdown of circPVT1 suppressed MTC cell proliferation, migration, and invasion. **a** & **b** The number of colonies formed in shNC transfected or shcircPVT1 transfected MTC cells. **c** Flow cytometry analysis of the number of transfected cells in G1, S, and G2 phases. **d** Flow cytometry analysis of cell apoptosis in MTC cells transfected with shNC or shcircPVT1. **e** & **f** Transwell assay assay to analyze migration and invasion abilities of transfected cells. **g** & **h** EMT-related protein levels in MTC cells transfected with shNC or shcircPVT1. * *P* < 0.05; ** *P* < 0.01; *** *P* < 0.001

**Fig. 6** c**i** rcPVT1 regulated MTC cells proliferation and apoptosis via miR-455-5p/CXCL12 axis. **a** Relative circPVT1 level in MTC cells transfected circPVT1 overexpressing vector. **b** & **c** The number of colonies formed in MTC cells transfected with circPVT1 overexpressing vector or miR-455-5p mimics or treated with CXCR4 antagonist AMD3100. **d** Flow cytometry analysis of the number of transfected cells in G1, S, and G2 phases. **e** Flow cytometry analysis of cell apoptosis in MTC cells transfected with circPVT1 overexpressing vector or miR-455-5p mimics or treated with CXCR4 antagonist AMD3100. * *P* < 0.05; ** *P* < 0.01; *** *P* < 0.001

These corrections do not affect the overall result or conclusion of the article. The original article has been corrected.
