# Supplementary figures and images for: circPVT1 regulates medullary thyroid cancer growth and metastasis by targeting miR-455-5p to activate CXCL12/CXCR4 signaling
Source: J Exp Clin Cancer Res. 2021 May 7;40:157. doi: 10.1186/s13046-021-01964-0 (PMC8106141; doi:10.1186/s13046-021-01964-0)

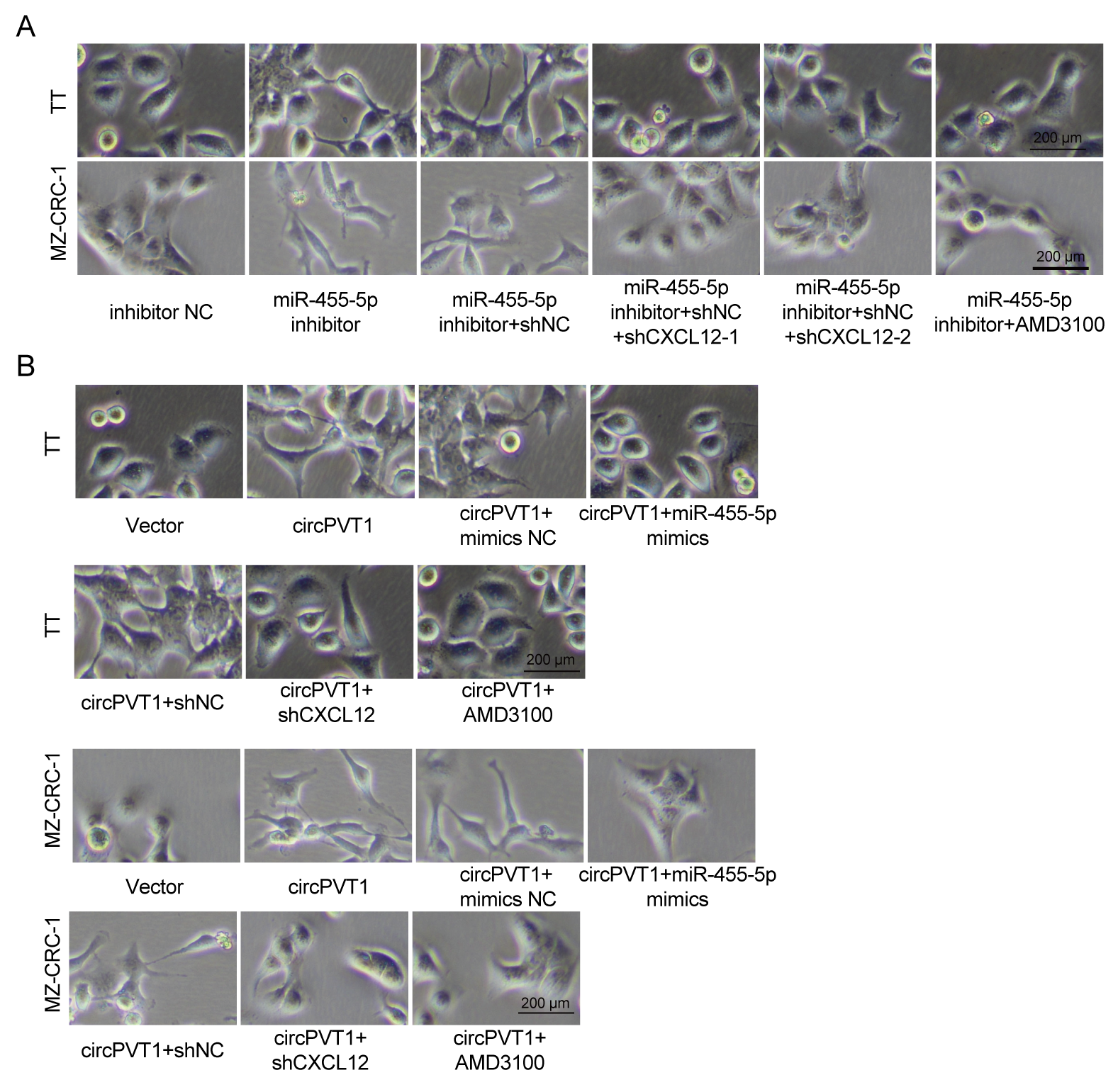

Supplement: Supplementary file 1 — Additional file 1: Figure S1. circPVT1/miR-455-5p regulated EMT viaCXCL12.(A&B) Morphologies of transfected cells. [file 13046_2021_1964_MOESM1_ESM.tif]
